# Perceptions of anti-smoking messages amongst high school students in Pakistan

**DOI:** 10.1186/1471-2458-11-117

**Published:** 2011-02-18

**Authors:** Syed MA Zaidi, Abdul L Bikak, Ayesha Shaheryar, Syed H Imam, Javaid A Khan

**Affiliations:** 1 Aga Khan University, Stadium Road, Karachi, Pakistan; 2 Section of Pulmonology, Department of Medicine, Aga Khan University, Stadium Road, Karachi, Pakistan

## Abstract

**Background:**

Surveys have provided evidence that tobacco use is widely prevalent amongst the youth in Pakistan. Several reviews have evaluated the effectiveness of various tobacco control programs, however, few have taken into account the perceptions of students themselves regarding these measures. The aim of this study was to determine the most effective anti-smoking messages that can be delivered to high-school students in Pakistan, based on their self-rated perceptions. It also aimed to assess the impact of pictorial/multi-media messages compared with written health warnings and to discover differences in perceptions of smokers to those of non-smokers to health warning messages.

**Methods:**

This study was carried out in five major cities of Pakistan in private English-medium schools. A presentation was delivered at each school that highlighted the well-established health consequences of smoking using both written health warnings and pictorial/multi-media health messages. Following the presentation, the participants filled out a graded questionnaire form, using which they rated the risk-factors and messages that they thought were most effective in stopping or preventing them from smoking. The Friedman test was used to rank responses to each of the questions in the form. The Wilcoxon Signed Rank test used to analyze the impact of pictorial/multi-media messages over written statements. The Mann Whitney U test was used to compare responses of smokers with those of non-smokers.

**Results:**

Picture of an oral cavity cancer, videos of a cancer patient using an electronic voice box and a patient on a ventilator, were perceived to be the most effective anti-smoking messages by students. Addiction, harming others through passive smoking and impact of smoking on disposable incomes were perceived to be less effective messages. Pictorial/multi-media messages were perceived to be more effective than written health warnings. Health warnings were perceived as less effective amongst smokers compared to non-smokers.

**Conclusion:**

Graphic pictorial/multi-media health warnings that depict cosmetic and functional distortions were perceived as effective anti-smoking messages by English-medium high school students in Pakistan. Smokers demonstrated greater resistance to health promotion messages compared with non-smokers. Targeted interventions for high school students may be beneficial.

## Background

Evidence indicates that most smokers initiate the habit before attaining adulthood[1]. In addition, young smokers and adolescents are more likely to develop nicotine dependence[2]. This acquires particular significance in developing countries with demographic patterns similar to Pakistan's, where the median age of the population is only 21 years[3]. Here, future trends of tobacco attributable mortality are likely to be influenced by current tobacco consumption and perceptions amongst the youth. This school-going age group comprises the largest segment of the population and is also the most susceptible towards experimentation with tobacco. Therefore, achieving tobacco control amongst this age group is critical for mitigating the public health consequences of this emerging epidemic.

Various surveys have provided evidence that tobacco use is widely prevalent amongst students and adolescents in the urban areas of Pakistan. The results of the Global Youth Tobacco Survey (GYTS) showed that tobacco use prevalence was 12.8% in males and 8.0% in females aged 13-15[4]. A study in Karachi evaluating smoking in males showed that prevalence increases to 19.2% in ages 15-17, 26.5% in ages 18-20 and reaches 65% in 21 years and above[5]. A survey in Karachi targeting adolescent female smoking showed a prevalence of 16.3% in the above 15 age group[6]. In addition shisha use is becoming increasingly popular in the student age group[7].

Whilst several reviews have evaluated the effectiveness of various tobacco control programs, few have taken into account the perceptions of students themselves regarding these measures. It is important to discover the factors that the youth considers as significant in either encouraging them to cease the habit or from initiating smoking in the first place. The current cigarettes pack warnings in the country that consist only of text, for example, may be less effective than pictorial warnings, as has been demonstrated elsewhere[8]. In addition, students that have already initiated smoking may be more resistant to anti-smoking messages. Such data is essential for review before effective health promotion advertisements, curricular material in textbooks and appropriate legislation can be introduced. Although a majority of anti-tobacco modalities are not specifically designed for the youth, there is evidence to suggest that such targeted interventions are highly effective ways of curtailing the tobacco epidemic [9,10].

Therefore, the aim of this study was to determine the most effective anti-smoking messages that can be delivered in order to reduce tobacco consumption amongst high-school students in Pakistan based on their own, self-rated perceptions and to highlight which risk-factors related to tobacco consumption did the students consider most significant in deterring them from smoking. We also aimed to test the hypothesis that pictorial/multi-media warnings will be more effective than text-only warnings and to discover whether there was any difference in the perceptions of smokers to those of non-smokers to these health messages.

## Methods

### Study Setting

This study was carried out in five cities of Pakistan, including Islamabad, Rawalpindi, Lahore, Faisalabad and Karachi, from January to February, 2010. These cities represent the major urban centers of Pakistan where the youth has access to tobacco products and is influenced by advertising. A minimum of two high schools from each city were identified for carrying out this study. These schools allowed convenient access to adolescents and were an appropriate setting to conduct the study.

### Study Design and Procedure

A Microsoft PowerPoint presentation was developed that highlighted some of the most important and well-established health consequences of smoking. These were derived and adapted from the US Surgeon General's report published in collaboration with the Centre for Disease Control[11]. The presentation consisted of slides that provided details and written warnings of each tobacco related illness. Following the health warnings of each specific illness, a slide utilizing different pictorial/multi-media aids was used to show the health outcome of the disease. These included a picture of smokers' lungs, pictures of oral cancer, a video of a person using an electronic voice box, a video of a patient on a ventilator and a video of a person paralyzed due to stroke. The pictures and multi-media aids were obtained from open-access websites such as *YouTube*. In addition, other harmful effects of tobacco such as addiction, social implications and dangers posed by secondhand smoke exposure were also highlighted in the presentation.

The presentation was delivered at each of the schools selected for the study. At the end of the presentation, the students were asked to fill out a graded questionnaire form, using which they rated the risk-factors and messages that they thought were most effective in stopping or preventing them from smoking (Table 1). The questionnaire form consisted of a total of 20 questions related to the anti-smoking messages with responses ranging from 1 to 5 (1 = Not at all, 2 = Unlikely, 3 = Unsure, 4 = Likely, 5 = Definitely). The demographics of the participants including age and gender were noted along with smoking status. Tobacco usage greater than once in the month preceding the administration of the questionnaire was taken as positive for both cigarette smoking and water-pipe smoking. This figure was adapted from the criteria used in the GYTS[4]. Prior ethical clearance was sought at the Aga Khan University.

**Table 1:** Mean rank scores of responses using the Friedman test

**Question**	**Mean Rank Score**
Did watching a picture of an oral cavity cancer convince you of the harmful effects of smoking?	11.04
Did watching a video of a person on a ventilator convince of you the harmful effects of smoking?	10.91
Did watching a video of a person using an electronic voice box convince you of the harmful effects of smoking?	10.77
Did watching a picture of cancerous lungs convince you of the harmful effects of smoking?	10.43
Did watching a video of a person recovering from stroke convince you of the harmful effects of smoking?	10.19
Smoking causes stroke, does knowing this risk stop you from smoking?	9.99
Smoking causes heart attacks, does knowing this risk stop you from smoking?	9.89
Smoking causes oral cancer, does knowing this risk stop you from smoking?	9.89
Smoking causes throat cancer, does knowing this risk stop you from smoking?	9.77
Smoking causes severe lung disease, does knowing this risk stop you from smoking?	9.57
Religious scholars consider smoking unlawful, does this stop you from smoking?	9.48
Smoking causes lung cancer, does knowing this risk stop you from smoking?	9.17
Does a ban on smoking in public areas stop you from smoking?	8.91
Passive smoking causes harm to others, does knowing this risk stop you from smoking?	8.61
Smoking leads to the use of other more dangerous drugs, does knowing this risk stop you from smoking?	8.16
Smoking is addictive, does knowing this risk stop you from smoking?	7.94
Smoking addiction adversely affects disposable incomes-Does knowing this risk stop you from smoking?	7.74

Friedman's p value < 0.001

### Sample

All of the schools enrolled in the study were private schools where the medium of instruction was in English. This was due to certain logistical and financial constraints of conducting the study in government-run schools such as, availability of multi-media projectors and back-up generators in case of power failures. A recent survey showed that a third of Pakistanis are educated in English-medium private schools and a further 15% are in English-medium government schools[12]. This sample may therefore not be representative of the remaining students belonging from a lower socio-economic group that are currently enrolled in other government schools or *madrassahs*. However, efforts are being made to convert these into English-medium facilities in the future[13]. The presentation was delivered to students in small groups consisting of approximately 40 students each. Both male and female students as well as current smokers and non-smokers were included. Approval from the faculty and the administration of the schools where the study was conducted was sought before delivering the presentation. The responsible faculty members were approached, briefed on the purpose of the study and were shown the details of the presentation for their approval. Student groups were then arranged by the schools' administration prior to the delivery of the presentation. The students were asked to sign a consent form that was included with the questionnaire for participating in the study.

### Data Analysis

The data was analyzed using Statistical Package for Social Sciences (SPSS v16.0). To compare responses to questions across the data set, the Friedman test for non-parametric data was utilized. This test was used to generate ranks between individual questions in the dataset. These were utilized to show which risk factors and multi-media aids adolescents considered as the most effective anti-smoking messages. To assess the impact of pictorial/multi-media health warnings, five questions pertaining to these were each paired with questions of their associated written text warnings (Table 2). The Wilcoxon Signed Ranks Test was utilized to assess whether there was any statistically significant difference in the responses to the questions within these pairs. The Mann Whitney U test was utilized to compare the responses of smokers to those of non-smokers. A p value of < 0.05 was taken as significant for each of the tests.

**Table 2:** Comparison of text warnings with multi-media warnings as deterrents from smoking

	**Text Warning**	**Multi-Media Warning**	**p-value**
**1**.	Smoking causes oral cancer	Picture of an oral cavity cancer	p < 0.001
**2**.	Smoking causes lung cancer	Picture of cancerous lungs	p < 0.001
**3**.	Smoking causes throat cancer	Video of a person using an electronic voice box	p < 0.001
**4**.	Smoking causes stroke	Video of a person recovering from stroke	p = 0.760
**5**.	Smoking causes severe lung disease	Video of a person a ventilator	p < 0.001

## Results

A total of 388 high school students were included in the study out which 245 were males and 142 were females. The mean age of the sample population was 17 with a standard deviation of 1.51. Out of the sample, a total of 97 (25.5%) identified themselves to be smokers out of which 70 were males (28.5% of males) and 27 were females (19% of females). A total of 150 (38.7%) participants answered positively for shisha smoking out of which 104 were males (42.5% of males) and 46 were females (32.4% of females).

Table 1 shows the mean rank scores generated using the Friedman test for the responses of each of the questions. "*Did watching a picture of an oral cavity cancer convince you of the harmful effects of smoking*," had the highest rank. *"Smoking addiction adversely affects disposable incomes-Does knowing this risk stop you from smoking," *ranked the lowest. The Friedman's p-value was < 0.001.

Table 2 shows the comparison of responses to questions regarding written health warnings with their associated multi-media messages. Responses were significantly greater for the pictorial/multi-media messages in each of the pairs except for *"Video of a person recovering from stroke," *which was not significantly different from the written statement.

The comparison of responses given by smokers to those of non-smokers yielded significantly lower scores (p < 0.01) by the former group across the question set.

Overall an encouraging response was received from the faculty and from the students to both the presentation and to the study in the schools that were visited. All of the students that were attending the presentations consented to be a part of the study. One of the schools approached, which was only for girls however, did not consent to the documentation of smoking prevalence of the students. The survey was not carried out at this school and only the presentation was delivered. An alternative school was subsequently selected for inclusion in the study.

## Discussion

Pakistan has taken a number of tangible steps towards reducing adolescent tobacco consumption in the country such as enforcing bans on tobacco advertising and underage sales. A recent decision by the Ministry of Health to introduce pictorial warnings on cigarette packs could also have a major impact[14]. However, for comprehensive enforcement of the Framework Convention for Tobacco Control (FCTC), the government will need to ensure that the warnings are rotated, are of appropriate size and are present on all packaging and labeling[15]. In addition, current tobacco control legislation is not directed against shisha smoking that is acquiring increasingly popularity amongst the youth[16].

Our results suggest that the effectiveness of the health messages could also be determined by the type of warning that is delivered. Graphic visual images, such as, pictures of oral cavity cancers were perceived to have the greatest impact in deterring students from smoking. Multi-media aids that conveyed messages students could relate to, both anatomically and functionally, ranked higher than the more commonly used pictures of a 'smoker's lungs,' that could perhaps not convey the health warning with a similar impact. Amongst these multi-media aids also included videos of a patient using an electronic larynx and a patient on a ventilator. These findings suggest that such multi-media aids may be effective advertisements for health promotion campaigns.

Our findings give further support to the use of pictorial and multi-media health warnings instead of warnings consisting only of text that were perceived to be less effective. This is particularly pertinent in countries with poor literacy rates such as Pakistan. In addition, cigarette pack warnings in the country are often in English, which is understood by a limited segment of the population, hence, obfuscating the necessary health promotion messages. Multi-media anti-smoking messages could therefore may improve awareness of the health consequences of smoking amongst the youth in Pakistan. Modifying label packaging to include graphic health warnings has been demonstrated as an effective means of reducing tobacco consumption and improving awareness of the health consequences of smoking in other countries within this age group[10,17-19].

The participants did not perceive the current ban on smoking in indoor public areas to be an impediment to smoking. This suggests that they are either unaware of the relevant legislation or that they do not believe the laws will be enforced and any violations will be dealt with. They also did not perceive harming others through second hand smoke to be a major deterring factor. These findings suggest that there is a substantial lack of awareness regarding the hazards of second hand smoke amongst adolescents. In addition, the low scores for responses to questions relating to addiction and to cigarettes as a 'gateway drug' also suggest a lack of awareness of the severity of these conditions. This is of significance in the school-going age group as addiction is cited as the commonest reason for failure of smoking cessation during adulthood[20].

Finally, those who identified themselves as smokers gave significantly lower responses to those of non-smokers across the question set. This suggests that the susceptibility to anti-smoking messages may decrease substantially once the habit has been initiated on a regular basis. Such early demonstration of intransigence to health promotion messages does not portend well for future smoking cessation during adulthood. This suggests that early, directed interventions aimed at students and adolescents may be beneficial as appropriate messages are delivered before the habit is initiated.

The study was limited by the fact it was carried out in private schools where the medium of instruction is English and the students belonged to relatively higher socio-economic group. This could explain why the impact of smoking on disposable incomes was not cited as a major deterring factor. This could however, be of greater relevance for adolescents belonging to a lower socio-economic group. Based on these findings, a follow-up study is now being carried in public schools where Urdu is the medium of instruction.

## Conclusion

Graphic visual images and multi-media aids that vividly depict cosmetic distortions and loss of normal organ function as outcomes of the diseases associated with smoking are perceived by high school students as the most effective modalities in deterring them from smoking. These aids, in the form of health warnings, health promotion campaigns and material in school curricula, may be useful as effective tobacco control modalities in developing countries with young populations. Students that have already initiated the habit may be more resistant to tobacco control messages, hence, early intervention may prove to be beneficial. In addition, lack of awareness of other hazardous effects of smoking such as addiction and secondhand smoke exposure needs to be addressed in Pakistan.

## Competing interests

The authors declare that they have no competing interests.

## Authors' contributions

SMAZ participated in the study design, manuscript writing, data collection and analysis. ALB participated in data analysis and manuscript writing. AS participated in data collection and analysis. SHI participated in data collection. JAK participated in study design, coordination and provided technical supervision.

All authors have read and approved the final manuscript.

## Pre-publication history

The pre-publication history for this paper can be accessed here:

http://www.biomedcentral.com/1471-2458/11/117/prepub
